# HOXA9/IRX1 expression pattern defines two subgroups of infant MLL-AF4-driven acute lymphoblastic leukemia

**DOI:** 10.1016/j.exphem.2020.10.002

**Published:** 2021-01

**Authors:** Vasiliki Symeonidou, Katrin Ottersbach

**Affiliations:** Centre for Regenerative Medicine, Institute for Regeneration and Repair, University of Edinburgh, Edinburgh, UK

## Abstract

Infant t(4;11) acute lymphoblastic leukemia is the most common leukemia in infant patients and has a highly aggressive nature. The patients have a dismal prognosis, which has not improved in more than a decade, suggesting that a better understanding of this disease is required. In the study described here, we analyzed two previously published RNA-sequencing data sets and gained further insights into the global transcriptomes of two known subgroups of this disease, which are characterized by the presence or absence of a homeobox gene expression signature. Specifically, we identified a remarkable mutually exclusive expression of the *HOXA9*/*HOXA10* and *IRX1* genes and termed the two subgroups iALL-HOXA9 and iALL-IRX1. This expression pattern is critical as it suggests that there is a fundamental difference between the two subgroups. Investigation of the transcriptomes of the two subgroups reveals a more aggressive nature for the iALL-IRX1 group, which is further supported by the fact that patients within this group have a worse prognosis and are also diagnosed at a younger age. This could be reflective of a developmentally earlier cell of origin for iALL-IRX1. Our analysis further uncovered critical differences between the two groups that may have an impact on treatment strategies. In summary, after a detailed investigation into the transcriptional profiles of iALL-HOXA9 and iALL-IRX1 patients, we highlight the importance of acknowledging that these two subgroups are different and that this is of clinical importance.

## Highlights

• We identified two sub-groups of infant MLL-AF4-driven ALL, iALL-HOXA9, and iALL-IRX1.
• The subgroups exhibit mutually exclusive expression of *HOXA9/HOXA10* and *IRX1*.
• The transcriptional profile of iALL-IRX1 patients revealed a more aggressive disease.
• The two subgroups exhibit different expression of potential therapeutic targets.

In the age of molecular medicine, transcriptional profiling of patients’ samples has become a vital component in improving our understanding of diseases. We now have the potential to dissect transcriptional variations among patients, identify unique components of their disease and provide customized treatment. This approach is particularly valuable for diseases that are rare and difficult to model as there is a scarcity of available information. A prominent example of such a disease is infant MLL-AF4-driven acute lymphoblastic leukemia (ALL). This devastating disease is known to arise in utero, and the patients have a poor prognosis 1, 2, 3, 4. With only a handful of patients diagnosed each year, a unique underlying biology, and a lack of accurate disease models, our understanding of this disease remains limited, which is reflected in the lack of progress in treating these patients [2]. Currently, we know that infant MLL-AF4-driven ALL can be divided further into two subgroups. Two classification systems can be found in the literature: one is based on the expression levels of the gene *HOXA9,* and the other on separating the patients based on the expression of genes in the HOXA and IRX family of proteins 5, 6, 7. This has been reported to be of clinical relevance as patients with HOXA9^high^/IRX^neg/low^ expression have a better prognosis than those with HOXA9^low^/IRX^pos^ expression 5, 6, 7. In this study, we set out to better understand these two subgroups of patients. To do this, we analyzed two previously published RNA-sequencing data sets derived from infant/pediatric patients with MLL-AF4-driven ALL [8,9].

## Methods

### RNA sequencing analysis pipelines

Raw reads were aligned with Kallisto (version 0.43.1) to GRCh38. The Bioconductor package Tximport was used to import transcript-level abundance, estimated counts, and transcript lengths (version 3.5) [10]. We initially performed batch correction using limma and filtered the samples for genes with low counts across samples. After filtering, determination of the expression level of each gene and differential expression analysis were performed using the DESeq2 pipeline (version 3.5) [11,12]. Genes were considered differentially expressed if they had an adjusted *p* value ≤0.1. Library pcaExplorer was used for PCA analysis [13]. Gene set enrichment analysis (GSEA) was performed using the GSEA Jana Desktop tool (version 4.1) [14,15]. R version 3.4.3 was used. It should be noted that before processing of the Andersson et al. [9] data set, Bam files were converted to Fastq with Samtools. GraphPad Prism version 7.0 was used.

## Results and discussion

**Figure 1:**
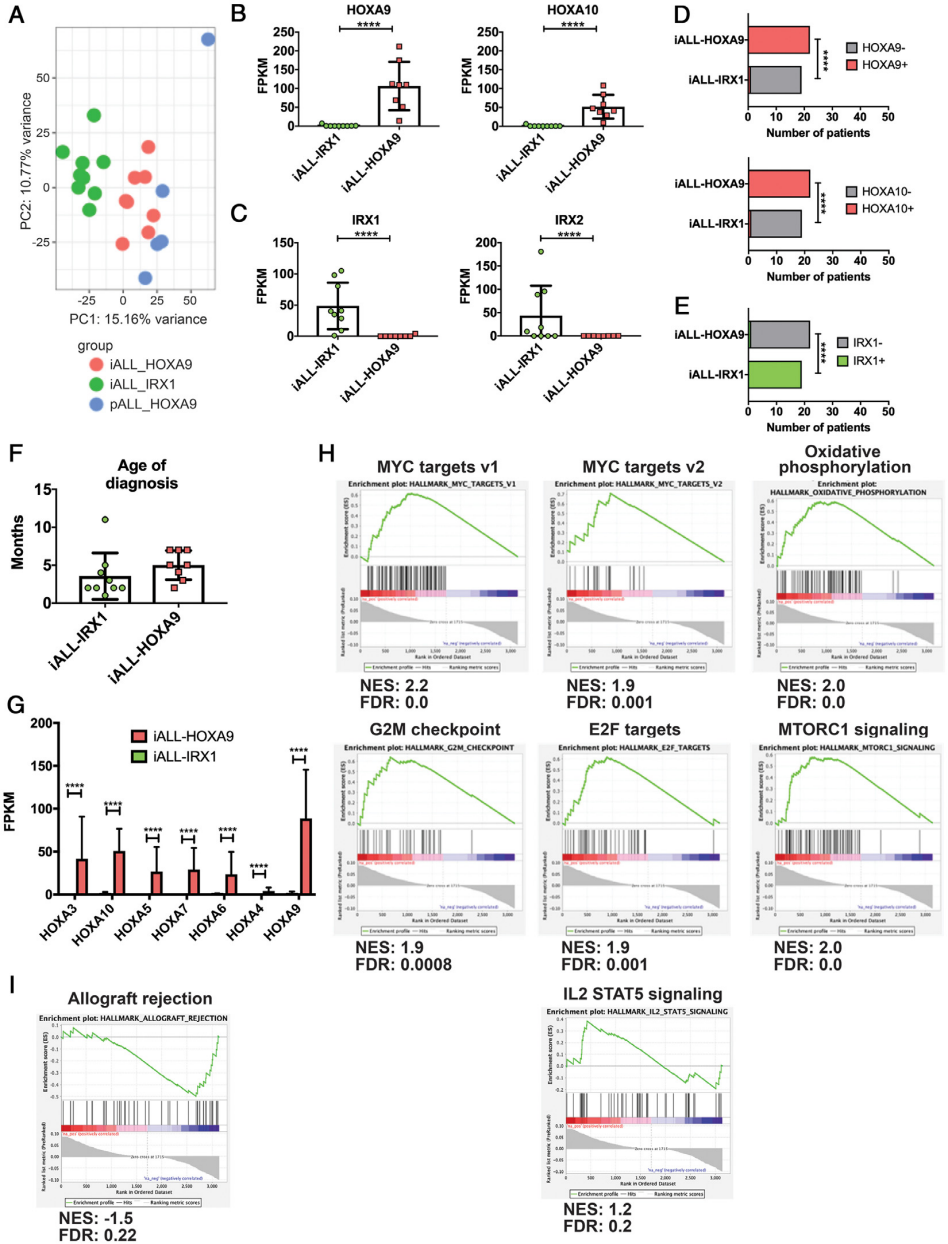
*HOXA9/HOXA10*–*IRX1* expression defines two subgroups of infant MLL-AF4-driven ALL (Andersson *et al.*[9] data set). **(A)** PCA of patients defined by *HOXA9/HOXA10* and *IRX1* expression and age at diagnosis. *Green* = infants with *IRX1* expression, *pink* = infants with *HOXA9/HOXA10* expression, *blue* = pediatric patients. All pediatric patients expressed *HOXA9*. **(B)***HOXA9* and *HOXA10* expression in the two subgroups. RNA Sequencing data are expressed as means ± SD; each *dot* represents a sample. **(C)***IRX1* and *IRX2* expression in the two subgroups. RNA sequencing data are expressed as means ± SD; each *dot* represents a sample. **(D)** Fisher's exact test comparing patient samples based on *HOXA9* and *HOXA10* expression levels (samples were deemed negative at FPKM <1 and positive at FPKM >1). **(E)** Fisher's exact test comparing patient samples based on *IRX1* expression levels (samples were deemed negative if FPKM was <1 and positive if FPKM was >1). **(F)** Age at diagnosis of patients with iALL-IRX1 and iALL-HOXA9. Data are expressed as means ± SD. Student's *t* test was performed. **(G)** Expression of HOXA cluster genes in the two subgroups. Data are expressed as means + SD. **(H,I)** Gene set enrichment analysis of iALL-IRX1 and iALL-HOXA9 patient samples. iALL-IRX1 patients **(H)** exhibit enrichment for MYC targets, oxidative phosphorylation, G2M checkpoints, E2F targets, MTORC1, and IL2 STAT5 signaling, whereas iALL-HOXA9 **(I)** patient samples exhibit enrichment for allograft rejection. *FPKM*=fragments per kilobase of transcript per million; *FDR*=false discovery rate; *NES*=normalized enrichment score. ****p < 0.0001.

We analyzed the RNA-sequencing data set of Andersson et al. [9], which contains data from 17 infant (<1 year) and 5 pediatric (11–18 years) patients with MLL-AF4-driven ALL. Principal component analysis (PCA) revealed that infant blasts formed two clusters (*pink* and *green* in Figure 1A). Intriguingly, blasts from pediatric patients (*blue*) clustered closely with one of the infant clusters. Investigation into the genes driving the clustering revealed *HOXA9, HOXA10* and *IRX1, IRX2* to be among the top PC1 loadings—in opposite directions. This confirmed previous publications reporting that clustering of the patients was driven by genes of the HOXA and IRX families [5,6]. To further investigate the expression pattern of these genes we performed Spearman's correlation test, which revealed an inverse correlation in the expression of *HOXA9, HOXA10,* and *IRX1,* but not *IRX2* (Figure 1B,C; Supplementary Table E1, online only, available at www.exphem.org). Although *IRX2* was one of the top differentially expressed genes, it was not uniformly upregulated in the HOXA9^low^/IRX^pos^ patients (Figure 1C). Furthermore, Fisher's exact test confirmed that the observed mutually exclusive expression was statistically significant (Figure 1D,E). These data suggest that the previously described infant ALL (iALL) HOXA9^low^/IRX^pos^ subgroup would be more accurately described as iALL-IRX1. It was also noteworthy that all pediatric patients expressed *HOXA9* and clustered closely with iALL-HOXA9. This may hint at the age of the patients at diagnosis as being another contributing factor to the division of infant patients into two subgroups, especially because a previous study suggested that expression patterns in infants change noticeably around the age of 90 days [5]. However, although patients in the iALL-IRX1 group appeared to be diagnosed at an earlier age, this did not reach statistical significance (Figure 1F). Investigation of the expression of all HOXA cluster genes revealed that they were uniquely upregulated in the iALL-HOXA9 subgroup, in line with previous reports of their coordinated expression (Figure 1G) [5,6,16].

GSEA of the genes differentially expressed between iALL-HOXA9 and iALL-IRX1 (Supplementary Table E2, online only, available at www.exphem.org) revealed an enrichment in MYC targets, as well as oxidative phosphorylation in the iALL-IRX1 patients (Figure 1H). The same subgroup also exhibited enrichment for proliferation pathways as exemplified by G2M checkpoints, E2F targets, and MTORC1 signaling upregulation (Figure 1H). This signature is indicative of the more aggressive nature of the blasts derived from these patients, which could explain their worse prognosis compared with iALL-HOXA9 patients. This is further supported by an additional enrichment of IL2 STAT5 signaling, a key component of core cancer pathways [17]. The top enriched pathway in iALL-HOXA9 patients was Allograft rejection indicative of an immune system-related response (Figure 1I).

**Figure 2:**
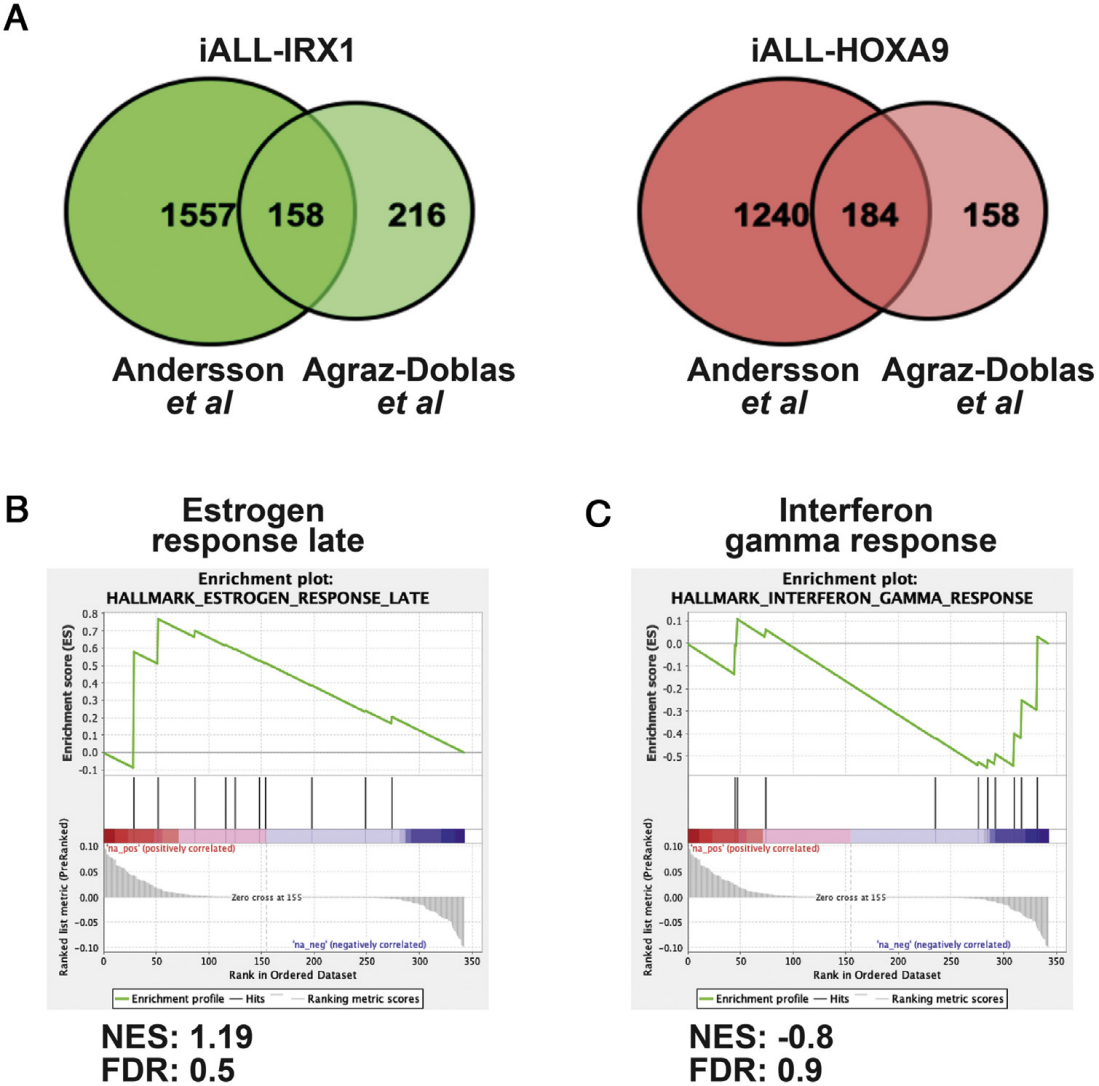
Gene set enrichment pathways coordinately upregulated in both RNA-sequencing experiments. **(A)** Venn diagram revealing genes upregulated in iALL-IRX1 and iALL-HOXA9 groups that are common in the two RNA-sequencing data sets (Andersson *et al.*[9] and Agraz-Doblas*et al.*[8]). **(B,C)** Gene set enrichment analysis of the genes common in the two RNA sequencing data sets. iALL-IRX1 **(B)** exhibited enrichment for Estrogen response late, whereas iALL-HOXA9 **(C)** exhibited enrichment in the Interferon gamma response. *FDR*=false discovery rate; *NES*=normalized enrichment score.

To further validate our data, we performed the same analysis with the data set of Agraz-Doblas et al. [8], which contains the transcriptome sequences of the blasts of 27 infant patients, and obtained similar results (Supplementary Figure E1A–F, Supplementary Tables E1 and E3, online only, available at www.exphem.org) [8]. To identify genes common to both data sets, we compared the genes differentially expressed between iALL-HOXA9 and iALL-IRX1 in both experiments (i.e., genes common between Supplementary Tables E2 and E3), which identified a total of 342 common genes (Figure 2A; Supplementary Table E4, online only, available at www.exphem.org). To obtain a general idea about these genes, we performed GSEA. There was an enrichment in Estrogen response late, which had been previously correlated with aggressive cancers, in the iALL-IRX1 group [18,19]. *HOXA9*-expressing blasts, on the other hand, exhibited an enrichment for Interferon gamma response, cementing our previous observation of an immune system response in these patients (Figure 2B,C). Both these signatures were present in the two individual RNA-sequencing experiments (Supplementary Figure 2A,B, online only, available at www.exphem.org).

It is intriguing that Homeobox genes *HOXA9, HOXA10,* and *IRX1* are inversely correlated in the two subgroups of infant patients with MLL-AF4-driven ALL. This mutually exclusive expression could be the result of the two subgroups having a different cell of origin. To investigate the expression pattern of these genes in human hematopoietic cells, we looked into previously published single-cell RNA-sequencing experiments with adult bone marrow and fetal liver-derived hematopoietic cells [20,21]. While *HOXA9* and *HOXA10* were expressed in hematopoietic stem and progenitor cells (both adult and fetal), *IRX1* exhibited very little expression in the hematopoietic system (Supplementary Figure E3A,B, online only, available at www.exphem.org) [20,21]. Interrogation of murine gastrulation and early organogenesis data sets revealed that *Irx1* was expressed predominantly in mesoderm, whereas *Hoxa9* and *Hoxa10* were expressed in hematoendothelial progenitors (Supplementary Figure E3C) [22].

**Figure 3:**
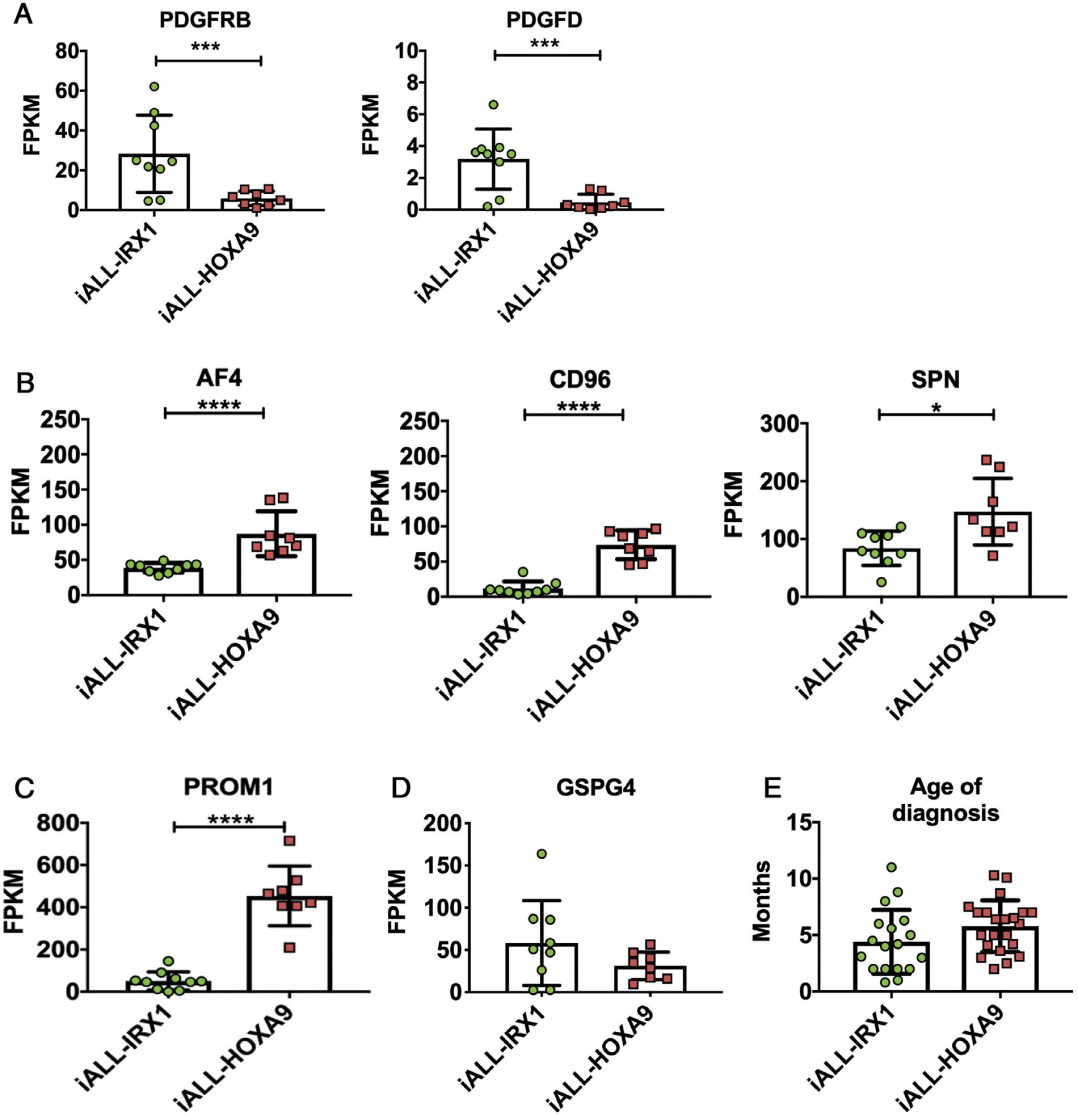
Genes differentially expressed between iALL-HOXA9 and iALL-IRX1 (Andersson *et al.*[9] data set). **(A)***PDGFRB* and *PDGFD* expression, **(B)***AF4 (AFF1), CD96,* and *SPN* expression, **(C)***PROM1* expression, and **(D)***GSPG4* expression in the two subgroups. RNA-sequencing data are expressed as means ± SD; each dot represents a sample. **(E)** Age at diagnosis of patients with iALL-IRX1 and iALL-HOXA9 (both data sets combined). ****p < 0.0001. ***p < 0.001. **p < 0.01. *p < 0.05.

The *IRX1* expression pattern could be indicative of iALL-IRX1 arising in a developmentally earlier cell type than iALL-HOXA9, which is supported by upregulation of genes such as *PDGFRB* and *PDGFD* in the iALL-IRX1 data set (Figure E3A; Supplementary Figure E4A, online only, available at www.exphem.org). Contrary to this, hematopoiesis-associated genes such as *AFF1 (AF4), CD96, SPN,* and *PROM1* are upregulated in the iALL-HOXA9 set (Figure 3B,C; Supplementary Figure E4B,C, Supplementary Table E4). Furthermore, as discussed above, patients with iALL-IRX1 appear to be diagnosed at a younger age as compared with iALL-HOXA9 patients (Figure 3E). As mesoderm has multiple progeny, including stromal cell components, it would not be surprising if MLL-AF4 was expressed in the bone marrow microenvironment of patients with iALL-IRX1. In fact, Menendez *et al.* [23] reported that a subset of bone marrow mesenchymal stromal cells of infant patients with MLL-AF4-driven ALL express the fusion gene. Although they do not specify whether the patients expressed *HOXA9* or *IRX1*, they do suggest that the disease could arise from a pre-hematopoietic precursor.

**Supplementary Fig. 1:**
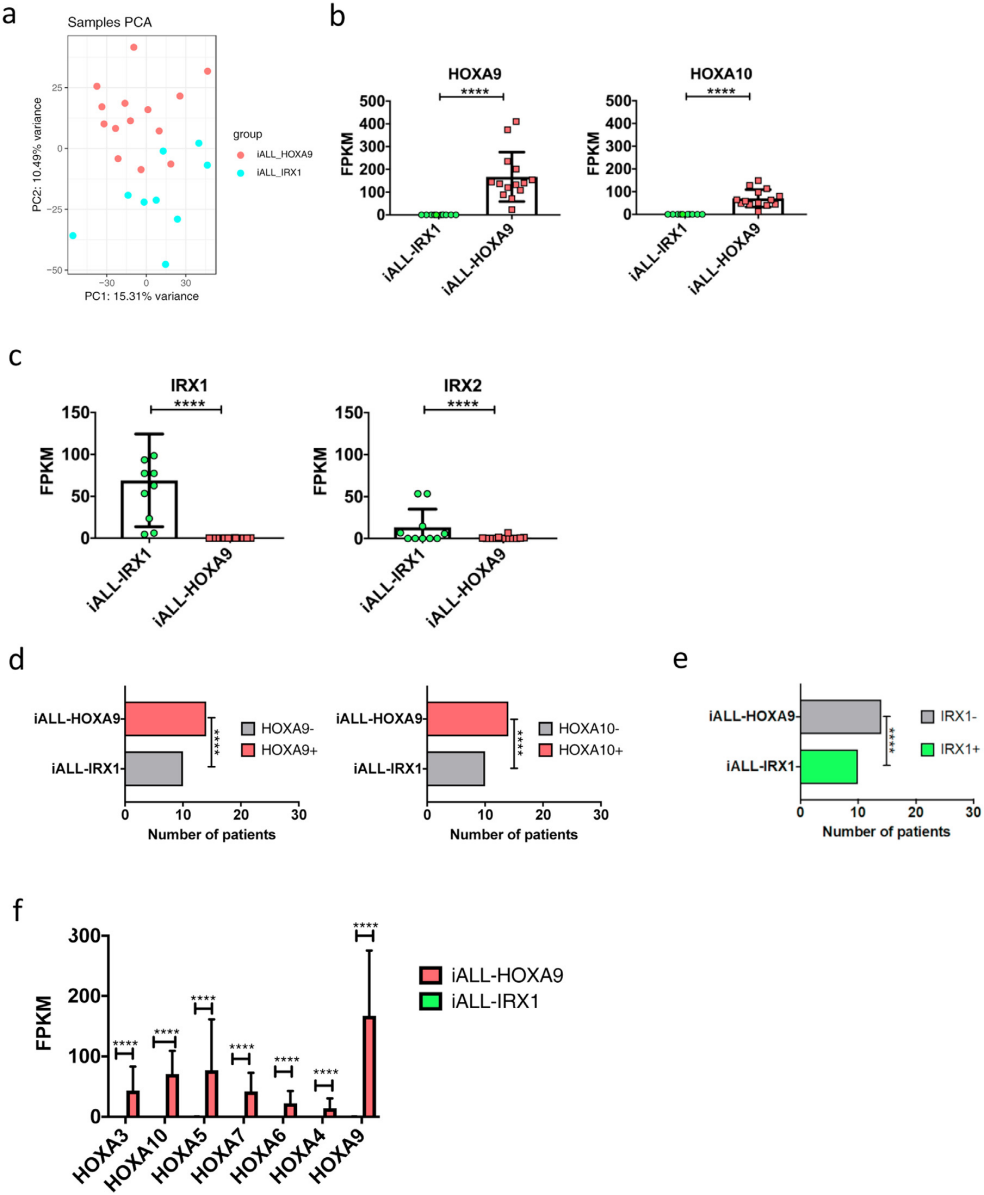
*HOXA9/HOXA10* – *IRX1* expression defines two subgroups of infant MLL-AF4-driven ALL (Agraz-Doblas *et al.* data set) **(A)** PCA of patients defined by *HOXA9/HOXA10* and *IRX1* expression. Pink = infants with *HOXA9/HOXA10* expression, blue = infants with *IRX1* expression. **(B)***HOXA9* and *HOXA10* expression in the 2 subgroups. RNA-sequencing data are shown as mean ±SD, each dot represents a sample. **(C)***IRX1* and *IRX2* expression in the 2 subgroups. RNA-sequencing data are shown as mean ±SD, each dot represents a sample. **(D)** Fisher's exact test comparing patient samples based on *HOXA9* and *HOXA10* expression levels (samples were deemed negative if FPKM<1 and positive if FPKM>1). **(E)** Fisher's exact test comparing patient samples based on *IRX1* expression levels (samples were deemed negative FPKM<1 and positive if FPKM>1). **(F)** Expression of *HOXA* cluster genes in the 2 subgroups. Data are shown as mean +SD. (It should be noted that 3 samples were removed from the analysis as two samples expressed both *HOXA9* and *IRX1,* while one of the samples did not express either *HOXA9* or *IRX1*. FPKM = Fragments Per Kilobase of transcript per Million; ****p<0.0001.

**Supplementary Fig. 2:**
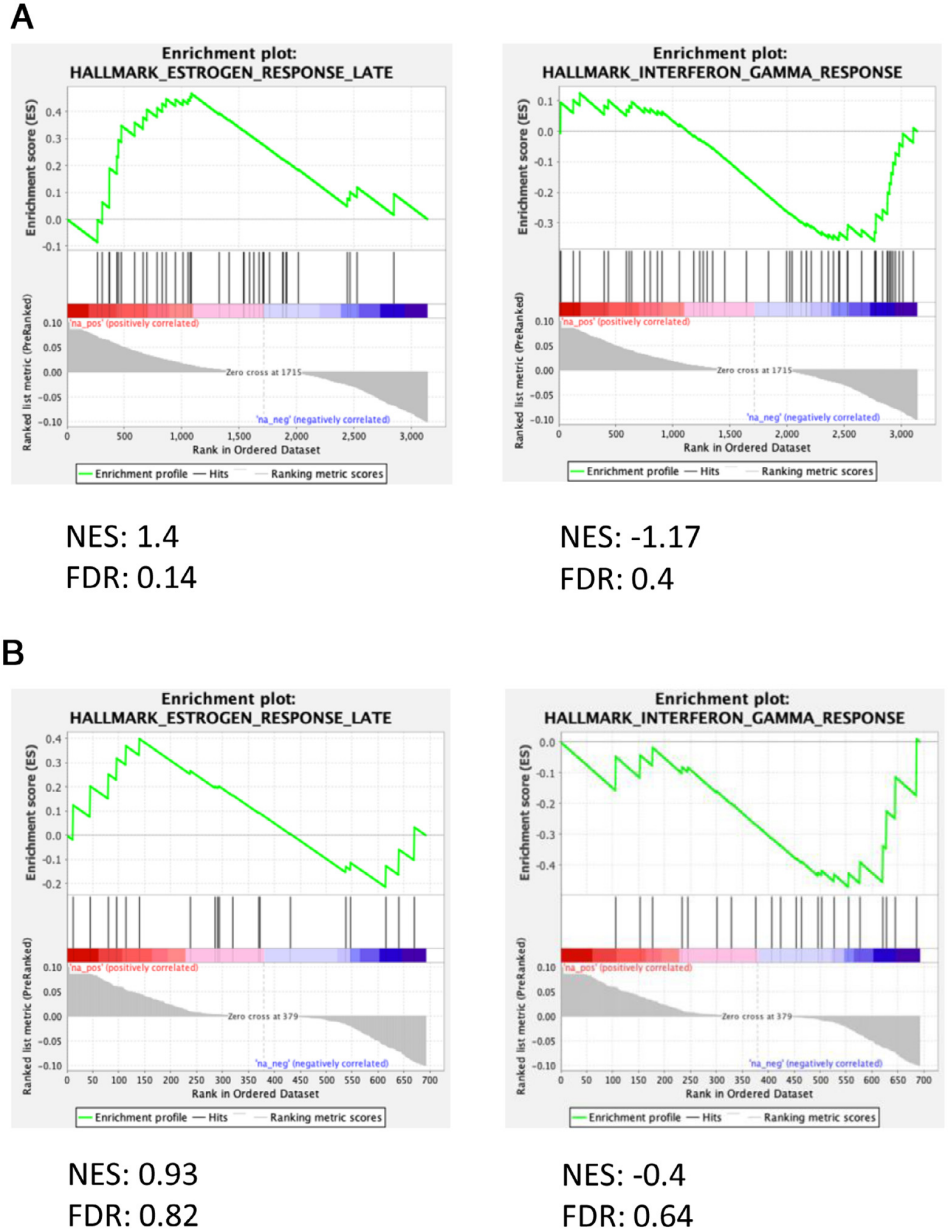
Gene set enrichment pathways co-ordinately upregulated in both RNA-sequencing experiments **(A)** Andersson *et al*. data and **(B)** Agraz-Doblas *et al.* data. iALL-IRX1 showed enrichment for Estrogen response late, whereas iALL-HOXA9 showed an enrichment in Interferon gamma response. FDR, false discovery rate; NES, normalized enrichment score.

**Supplementary Fig. 3:**
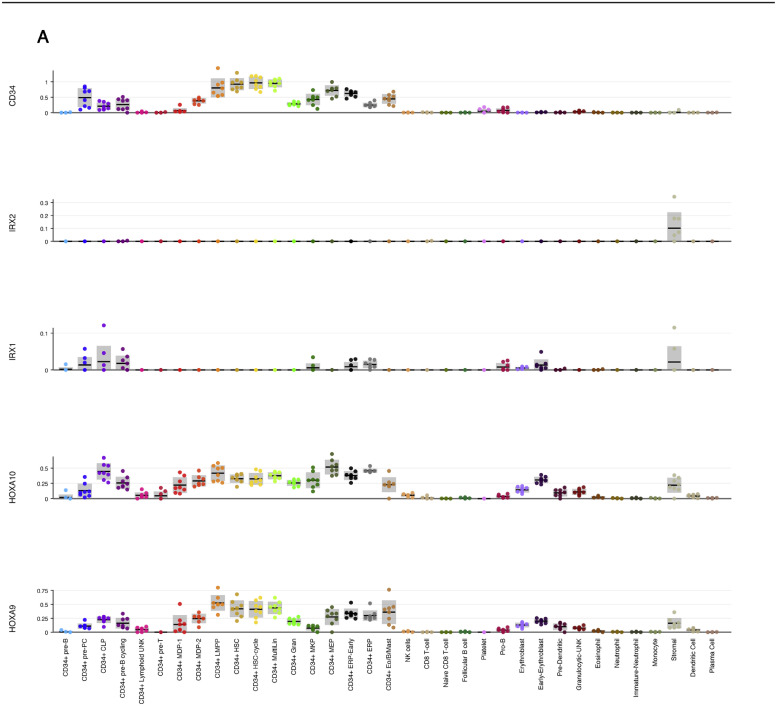
Continued.

**Supplementary Fig. 3:**
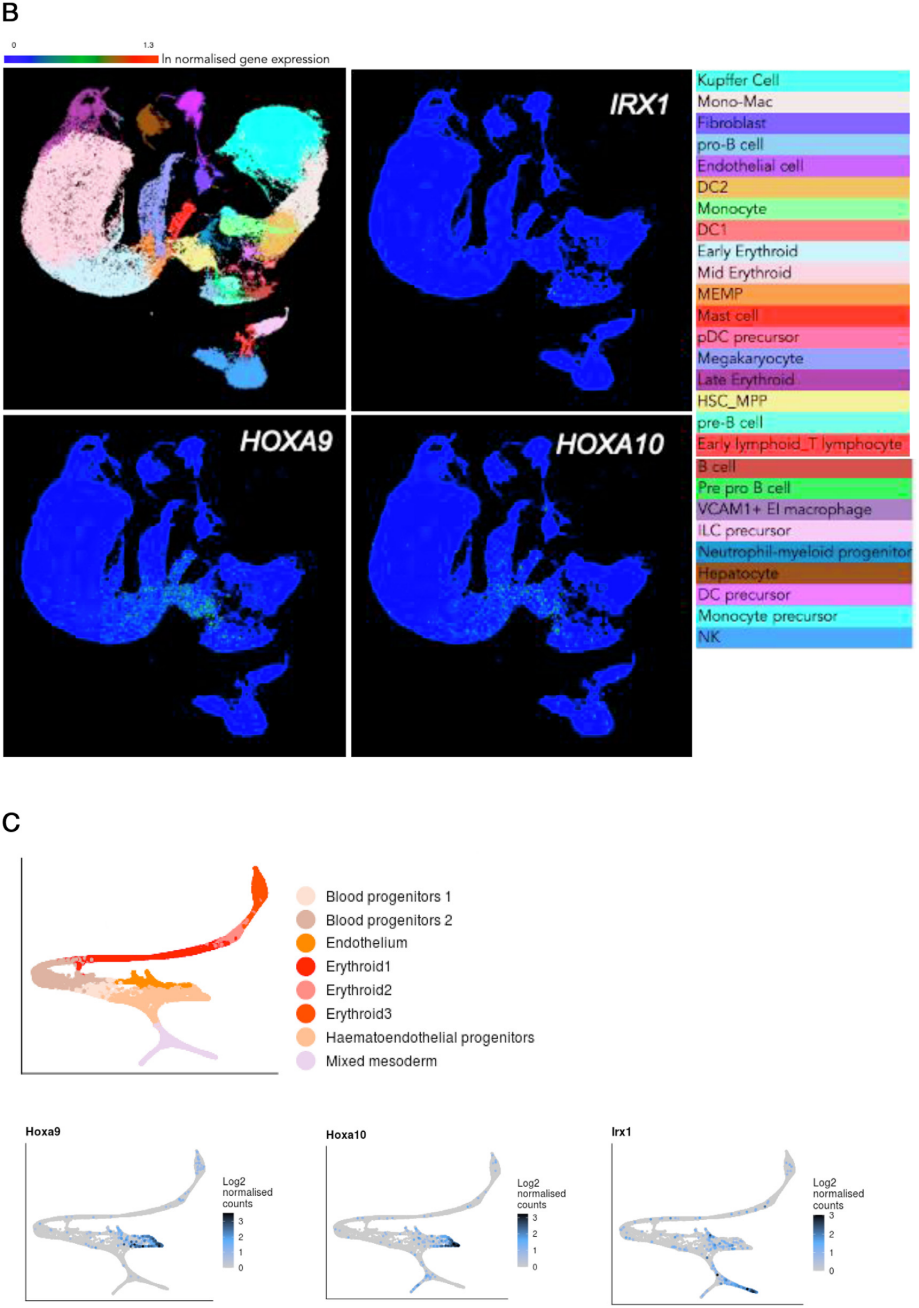
*HOXA9/HOXA10/IRX1* expression pattern in previously published single cell RNA-sequencing data sets. **(A)***HOXA9, HOXA10* and *IRX1* expression in human adult bone marrow derived cells. Data obtained from Human Cell Atlas - Bone Marrow (www.altanalyze.org/ICGS/HCA/splash.php). **(B)***HOXA9, HOXA10* and *IRX1* expression in human fetal liver-derived cells. Data obtained from Human Cell Atlas - Developmental (www.developmentcellatlas.ncl.ac.uk). (**C**) *Hoxa9, Hoxa10* and *Irx1* expression in mouse gastrulation and early organogenesis (www.marionilab.cruk.cam.ac.uk/MouseGastrulation2018/)

**Supplementary Fig. 4:**
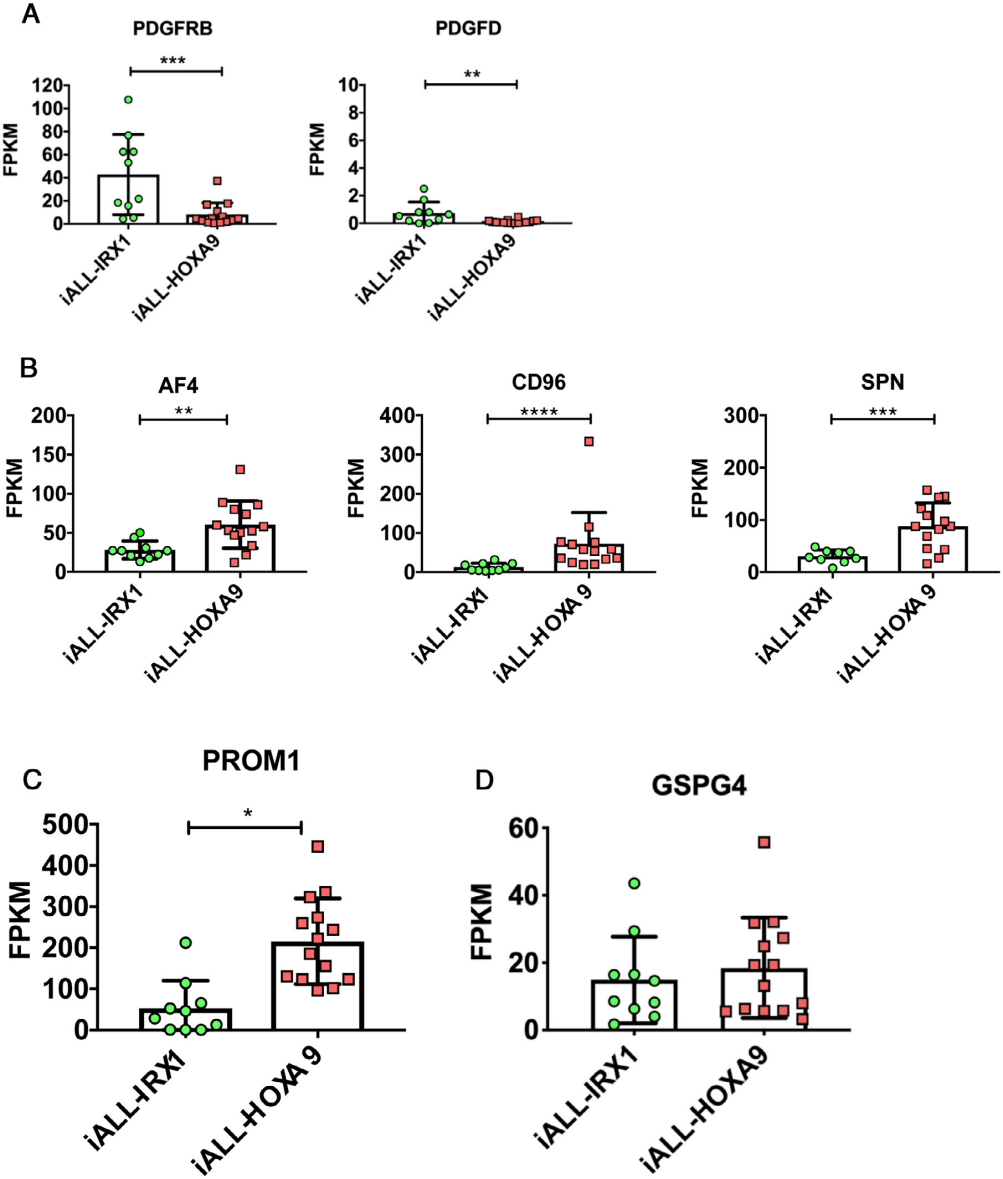
Genes differentially expressed between iALL-HOXA9 and iALL-IRX1 (Agraz-Doblas *et al*. dataset) **(A)***PDGFRB* and *PDGFD* expression in the 2 subgroups. RNA-sequencing data are shown as mean ±SD, each dot represents a sample. (B) *AF4, CD96* and *SPN* expression in the 2 subgroups. RNA-sequencing data are shown as mean ±SD, each dot represents a sample. **(C)***PROM1* expression in the 2 subgroups. RNA-sequencing data are shown as mean ±SD, each dot represents a sample. **(D)***GSPG4* expression in the 2 subgroups. RNA-sequencing data are shown as mean ±SD, each dot represents a sample. ****p<0.0001, ***p<0.001, **p<0.01, *p<0.05

The differences between the two groups may also have an impact on treatment options for these patients. For example, PROM1 (CD133), which has recently been suggested to be a target for MLL-AF4+ patients, was specifically upregulated in the iALL-HOXA9 subgroup, with lower expression in the iALL-IRX1 group of patients (Figure 3C; Supplementary Figure E4C) [24,25]. Treating iALL-IRX1 patients with CD133-directed CAR-T cells might therefore not be as effective as for iALL-HOXA9 patients. Another recently described therapeutic target for MLL-rearranged patients is GSPG4 (NG2), which is expressed at similar levels in both iALL-HOXA9 and iALL-IRX1 patients—albeit at lower levels than PROM1, suggesting that the outcome of this treatment could be similar for both subgroups (Figure 3D; Supplementary Figure 4D) [26].

One key point about the two subgroups of patients with MLL-AF4-driven ALL is that the majority of information we have gathered about this disease to date is derived from mouse models and cell lines, such as SEM, that express *HOXA9*. Therefore, it is important to replicate experiments in models and cell lines that mirror the iALL-IRX1 disease. We believe this would be critical when selecting therapeutic regimes for these patients, as exemplified by PROM1. We believe that future studies should therefore consider the *HOXA9* and *IRX1* expression status of infant ALL patients.

## Conflict of interest disclosure

The authors declare no competing interests.

## Appendix Supplementary materials

Image, application 1

## Footnotes

Supplementary material associated with this article can be found in the online version at https://doi.org/10.1016/j.exphem.2020.10.002.
